# Consensus statements and recommendations on the management of mild‐to‐moderate gastroesophageal reflux disease in the Southeast Asian region

**DOI:** 10.1002/jgh3.12602

**Published:** 2021-07-31

**Authors:** Khean‐Lee Goh, Yeong‐Yeh Lee, Somchai Leelakusolvong, Dadang Makmun, Monthira Maneerattanaporn, Duc Trong Quach, Raja Affendi Raja Ali, Jose D Sollano, Van Huy Tran, Reuben Kong‐Min Wong

**Affiliations:** ^1^ Department of Medicine University of Malaya Kuala Lumpur Malaysia; ^2^ Department of Medicine, School of Medical Sciences Universiti Sains Malaysia Kota Bharu Malaysia; ^3^ Division of Gastroenterology, Faculty of Medicine, Siriraj Hospital Mahidol University Bangkok Thailand; ^4^ Division of Gastroenterology, Department of Internal Medicine Faculty of Medicine Universitas Indonesia/Cipto Mangunkusumo National General Hospital Jakarta Indonesia; ^5^ Department of Internal Medicine University of Medicine and Pharmacy Ho Chi Minh City Vietnam; ^6^ Gastroenterology Unit, Department of Medicine and Gut Research Group, Faculty of Medicine Universiti Kebangsaan Malaysia Bangi Malaysia; ^7^ Department of Gastroenterology University of Santo Tomas Manila Philippines; ^8^ Department of Gastroenterology, Hue University of Medicine and Pharmacy Hue University Hue City Vietnam; ^9^ Department of Medicine, Faculty of Medicine National University of Singapore Singapore

**Keywords:** alginates, consensus recommendations, mild‐to‐moderate gastroesophageal reflux disease, proton‐pump inhibitors

## Abstract

This paper reports the proceedings from the first consensus meeting on the management of mild‐to‐moderate gastroesophageal reflux disease (GERD) in the Southeast Asian (SEA) region. Seventeen statements were drawn up by a steering committee that focused on epidemiology, mechanism of action, diagnostic investigations, and treatment. Voting on the recommendations used the Delphi method with two rounds of voting among the 10 panel members. The consensus panel agreed that GERD is mostly a mild disease in the SEA region with predominantly non‐erosive reflux disease (NERD). Complicated GERD and Barrett's esophagus are infrequently seen. The panel recommended endoscopy in patients with alarm or refractory symptoms but cautioned that the incidence of gastric cancer is higher in SEA. pH and impedance measurements were not recommended for routine assessment. The acid pocket is recognized as an important pathogenic factor in GERD. Lifestyle measures such as weight reduction, avoidance of smoking, reduction of alcohol intake, and elevation of the head of the bed were recommended but strict avoidance of specific foods or drinks was not. Alginates was recommended as the first‐line treatment for patients with mild‐to‐moderate GERD while recognizing that proton‐pump inhibitors (PPIs) remained the mainstay of treatment of GERD. The use of alginates was also recommended as adjunctive therapy when GERD symptoms were only partially responsive to PPIs.

## Graphical abstract

Algorithm for the management of mild‐to‐moderate gastroesophageal reflux disease in the Southeast Asian region derived from the consensus statements and recommendations of an expert panel.

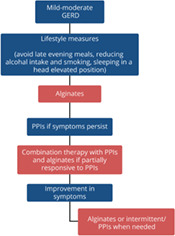

## Introduction

Gastroesophageal reflux disease (GERD) is defined as a disorder in which gastric contents reflux recurrently into the esophagus, causing troublesome symptoms and/or complications.^1^ It may present with symptoms or presence of reflux esophagitis at endoscopy. Traditionally, GERD has been considered an uncommon disease among Asian patients.^2^, ^3^ In a comparative study by Kang and Ho^4^ and in another study by Mahadeva *et al*.,^5^ GERD was diagnosed much more frequently in Western patients than in Asian patients. However, in a later review, Goh provided evidence that GERD has become more prevalent in the Asia‐Pacific region and is an emerging and important disease amongst Asians.^6^ More recent studies support this notion that GERD has indeed become more frequent in the Asia‐Pacific region.^7^, ^8^

However, in clinical practice, in the SEA region, GERD is generally mild. The majority of cases are nonerosive reflux disease (NERD) and most patients with reflux esophagitis present with milder grades of esophagitis.^9^ The diagnosis of “mild‐to‐moderate GERD” is made based on symptoms and is defined as awareness of reflux symptoms, but easily tolerated (mild) and discomforting reflux symptoms sufficient to cause interference with normal activities but is tolerable (moderate).^10^, ^11^

To address various issues with GERD in the region, three Asia‐Pacific consensus meetings have been held and their proceedings published.^1^, ^12^, ^13^ The SEA region represents a region where people share a common sociocultural and environmental background despite having a multiracial composition. There have, however, been no consensus meetings on GERD, specifically tailored to the SEA region. In this paper, we present our proceedings from a consensus meeting, focusing on the management of mild‐to‐moderate GERD in the SEA region.

## Methods

### Consensus development process

The consensus process was developed and coordinated by a steering committee, comprising of Khean‐Lee Goh, Reuben Kong‐Min Wong, Yeong‐Yeh Lee, and Somchai Leelakusolvong. Four areas were discussed: epidemiology, mechanism of action, diagnostic investigations, and treatment. Panel members were chosen from “key opinion leaders” from the Southeast Asian region, including Vietnam, Thailand, Philippines, Singapore, Indonesia, and Malaysia. The consensus process consisted of a series of virtual meetings that were held among panel members from December 2020 to February 2021.

The consensus statements were drawn up by the steering committee and modified after discussion with the panel members. These statements together with supporting evidence were circulated to all panel members. The Delphi method was used for consensus development. Voting by each faculty member was done anonymously through an electronic system. Two rounds of voting were carried out. During the voting process, panel members were asked to indicate their recommendation, level of evidence, and their agreement. After the first round of voting, panel members were given an opportunity to make comments for further modifications of statements and on supporting evidence that were provided. The steering committee perused the comments and made appropriate modification of statements and supporting evidence.

The revised statements were then circulated electronically for a second and final round of voting and the results and consensus agreement are presented in this article. For consensus agreement, each panel member was asked to indicate one of the following levels of agreement: strongly agree, agree with minor reservation, agree with major reservation, disagree with minor reservation, disagree with major reservation, and strongly disagree. If the member's vote was other than strongly agree or agree with minor reservation, they were asked to give the reasons for reservation or disagreement. Consensus level was predefined as ≥80% of the sum of the votes of strongly agree plus agree with minor reservation. Recommendation grade and evidence level were based on the GRADE system^14^ (Table 1). For the statements on “Epidemiology” (Statements 1–5) and “Mechanisms of disease” (Statements 6–8), no recommendations were made and therefore, the “Grade of recommendation” were deemed “not applicable.”

**Table 1:** Categorization of quality of evidence, strength of recommendations, and consensus agreement^14^

Level/grade	Description
Quality of evidence	
High	Further research is very unlikely to change our confidence in the estimate of effect
Moderate	Further research is likely to have an important impact on our confidence in the estimate of effect and may change the estimate
Low	Further research is very likely to have an important impact on our confidence in the estimate of effect and is likely to change the estimate
Very low	Any estimate of effect is very uncertain
Strength of recommendations	
Strong	When the desirable effects of an intervention clearly outweigh the undesirable effects, or clearly do not, guideline panels
Weak	When the trade‐offs are less certain‐either because of low quality evidence or because evidence suggests that desirable and undesirable effects are closely balanced
Agreement for consensus^†^	
1.	Strongly agree
2.	Agree with minor reservation
3.	Agree with major reservation
4.	Disagree with minor reservation
5.	Disagree with major reservation
6.	Strongly disagree

^†^ Consensus level was defined as ≥80% of the sum of the votes of strongly agree plus agree with minor reservation.

## Results

### Consensus statements

Section 1: EPIDEMIOLOGY
Statement 1: The incidence of GERD is increasing in the Southeast Asian region
Grade of Recommendation: not applicable
Evidence level: Moderate
Consensus level: 100% (Strongly agree—100%)

There are reports that clearly show the rise in the prevalence rates of GERD symptoms over time in the Southeast Asian region.^6^, ^8^, ^15^, ^16^, ^17^ Many other studies have shown significant increase in the prevalence of erosive esophagitis with time in this region.^18^, ^19^, ^20^, ^21^

A systematic review published in 2011 found the prevalence of symptom‐based GERD in East Asia to be 2.5–4.8% before 2005 and 5.2–8.5% between 2005 and 2010 (based on population‐based studies). The prevalence was found to be higher in Southeast and Western Asia (6.3–18.3% after 2005). There was a substantial increase in the prevalence of endoscopic reflux esophagitis in East Asia from the year 2000 to 2010 (3.4–5.0% before 2000 to 4.3–15.7% after 2005).^15^ Another systematic review published in 2014 found the prevalence of GERD in East Asia to be ranging between 2.5 and 7.8%.^8^

Statement 2: The majority of cases of GERD are NERD
Grade of Recommendation: not applicable
Evidence level: High
Consensus level: 100% (Strongly agree—80%; Agree with minor reservation—20%)

Gastroesophageal reflux disease can broadly be divided into two categories: NERD and erosive reflux disease (ERD).^22^ Overall, NERD accounts for majority of GERD patients (50–85%).^23^ Furthermore, NERD appears to be the most common form of GERD among patients in Asia‐Pacific region (78–93% of all reflux disease).^6^, ^9^, ^13^

Statement 3: Complications of GERD such as bleeding and strictures are uncommon in Asian patients
Grade of Recommendation: not applicable
Evidence level: Moderate
Consensus level: 100% (Strongly agree—70%; Agree with minor reservation—30%)

Reports on complications of GERD are limited. Wong *et al*. reported strictures in only 0.08% of patients in a study from Hong Kong.^24^ In a more recent study by Sakaguchi *et al*. from Japan bleeding and strictures were reported in 4.8 and 2.6% of patients with ERD, respectively, and both bleeding and strictures in 0.8% of patients.^25^

Statement 4: The prevalence of Barrett's esophagus is low in the region
Grade of Recommendation: not applicable
Evidence level: High
Consensus level: 90% (Strongly agree—80%; Agree with minor reservation—10%; Agree with major reservation—10%)

A systematic review including 51 studies (*n* =   453,147), mainly from Eastern Asia, evaluated the prevalence of Barrett's esophagus in Asian countries.^26^ The pooled prevalence of endoscopic Barrett's esophagus (23 studies) was 7.8% and of histologically‐confirmed Barrett's esophagus (28 studies) was 1.3% and most (82.1%) were short‐segment (<3 cm). In East‐Asian countries, between 1991 and 2014, there was a trend towards an increase in the prevalence of Barrett's esophagus.^26^ In a recent cross‐sectional study conducted in Vietnam on outpatients who underwent upper gastroscopy, biopsies were performed in endoscopically‐suspected esophageal metaplasia. The prevalence of Barrett's esophagus was 2.4% and most were short‐segment.^27^

Statement 5: Many cases of NERD have poor response to PPIs or have breakthrough symptoms while on PPI treatment
Grade of Recommendation: not applicable
Evidence level: High
Consensus level: 100% (Strongly agree—80%; Agree with minor reservation—20%)

There are many patients with GERD who do not respond to PPIs because of weakly acidic reflux, duodenogastroesophageal reflux, residual acid reflux due to inadequate control of the acid pocket, presence of hiatus hernia, and functional heartburn.^28^ In an Asian‐Pacific survey, many patients continued to experience GERD‐associated symptoms despite being on PPI therapy.^29^ The reasons for the poor response may be due to poor compliance, incorrect timing, and dosing of PPIs. A large proportion of patients may also have functional esophageal symptoms and/or esophageal hypersensitivity.^30^ Acid suppressive therapy will have limited success of symptom improvement in the presence of functional symptoms and/or esophageal hypersensitivity. NERD patients with hypersensitive esophagus demonstrate only partial or no response to PPI treatment.^31^

Most NERD patients have a significantly lower response rate to PPI therapy, and consequently they constitute the majority of the refractory heartburn group.^32^ In a systematic review, the overall PPI symptomatic response rate was reported at 36.7% in NERD and 55.5% in erosive esophagitis patients.^33^

Section 2: MECHANISM OF DISEASE
Statement 6: The acid pocket is a physiological finding in the human stomach formed after a meal
Grade of Recommendation: not applicable
Evidence level: High
Consensus level: 100% (Strongly agree—80%; Agree with minor reservation—20%)

A pH measurement study by Fletcher *et al*. showed the presence of an unbuffered acidic region near the gastroesophageal squamocolumnar junction during the post‐prandial period. This region escaped the buffering effect of meals, remaining highly acidic (median pH 1.6) compared with the body of the stomach (pH 4.7) and the authors coined the term acid pocket to denote this phenomenon.^34^ Subsequent studies have confirmed the existence of this acid pocket post‐prandially.^35^, ^36^, ^37^ A randomized, double‐blind, placebo‐controlled study by Vo *et al*. confirmed the presence of post‐prandial acid pockets as unbuffered zone of acidity in the proximal stomach/gastroesophageal junction region that were decreased in number, length, and magnitude after rabeprazole therapy.^38^

Statement 7: The acid pocket plays an important role in causing acid reflux
Grade of recommendation: not applicable
Evidence level: High
Consensus level: 100% (Strongly agree—80%; Agree with minor reservation—20%)

The acid pocket is a physiologic phenomenon that occurs in both healthy individuals and GERD patients.^39^ The acid pocket has been postulated to be the source of acidic refluxate, through shortening of lower esophageal sphincter (LES) after a meal^34^ or during transient hiatus herniation.^40^ Clarke *et al*. described that a region of unbuffered post‐prandial acid just below the gastroesophageal junction was more frequent and longer in severe reflux patients than in healthy subjects.^36^ Following meals, the acid pocket extends close to or across the squamocolumnar junction causing a short‐segment reflux that may be the reason for high incidence of inflammation and metaplasia at the gastroesophageal junction.^36^ PPIs reduce the acidity of the post‐prandial acid pocket, leading to less acidic refluxate, supporting the concept of the acid pocket as a source for post‐prandial acid reflux in patients with GERD taking PPI treatment.^41^

It was observed that acid reflux was more frequent in GERD and obese patients than in healthy individuals possibly due to concomitant presence of hiatal hernia. It is unclear why this happens, but among obese individuals, there is partial hiatus hernia from increased abdominal pressure, increasing short‐segment reflux, and delay in clearance of reflux.^42^ When hiatus hernia is present, the acid pocket is located above the diaphragm, thereby making it very easy for acid to reflux into the lower esophagus. A possible explanation is that patients with GERD commonly have a hiatal hernia.^39^

Hiatus hernia contributes to the pathophysiology of reflux disease by reducing the LES pressure, thereby impairing its function.^43^ In patients with small hiatal hernia, intermittent reduction of hernia occurs frequently; however, spatial separation of the diaphragm and LES results in increased occurrence of gastroesophageal reflux.^44^ A study by Beaumont *et al*. concluded that the enlarged acid pocket and its presence above the diaphragm, especially in patients with hiatal hernia, is a major risk factor for acidic reflux during transient lower esophageal relaxations (TLESRs).^45^

Statement 8: Alginate compounds form a raft above the acid pocket and prevents reflux of acid and nonacidic contents (volume reflux) of the stomach into the lower esophagus
Grade of recommendation: not applicable
Evidence level: Moderate
Consensus level: 100% (Strongly agree—90%; Agree with minor reservation—10%)

Sodium alginate reaction with acid produces a low‐density viscous gel (“raft”) that floats on top of the stomach forming a physical barrier that suppresses the gastric reflux. Alginate raft can also cover the acid pocket to reduce or prevent post‐prandial acid reflux.^46^A randomized, controlled, double‐blind, cross‐over clinical study in 20 patients referred for investigation of reflux symptoms revealed a decline in proximal reflux events with raft‐forming alginate (Gaviscon Advance) compared with non‐raft forming antacid (Milk of Magnesia) (10.5 *vs* 13.9).^47^ This provides prima facie evidence for the efficacy of alginates, and we look forward to larger studies to solidify their use in practice.

Section 3: DIAGNOSTIC INVESTIGATIONS
Statement 9: Endoscopy is indicated when patients present with alarm or refractory symptoms
Grade of recommendation: Strong
Evidence level: Moderate
Consensus level: 100% (Strongly agree—90%; Agree with minor reservation—10%)

Endoscopy at presentation should be considered in patients who have symptoms suggestive of complicated disease (for example: dysphagia, unintentional weight loss, hematemesis) or those with multiple risk factors for Barrett's esophagus.^48^ Additionally, in areas of the Southeast Asian region, where the incidence and prevalence of gastric cancer and peptic ulcer disease are high, there has to be a high index of suspicion for these diseases. Patients presenting with upper abdominal symptoms of recent onset, those with a family history of gastric cancer, and those with mild weight loss and anemia should undergo a gastroscopy. In a study from Hong Kong, Wu *et al*. had emphasized that empirical treatment for “typical” reflux symptoms was inappropriate in their population with high *Helicobacter pylori* prevalence.^49^ In their study, they found a high proportion of patients with peptic ulcer disease (18%). In another study from Hong Kong, there was a higher prevalence of upper gastrointestinal tract cancers in patients presenting with dyspepsia.^50^ In regions with high prevalence like Vietnam, early‐onset gastric cancer is not rare and only about two‐thirds of patients with advanced lesions have alarm features.^51^

Statement 10: pH monitoring and impedance testing are not necessary in the routine management of mild‐to‐moderate GERD
Grade of recommendation: Strong
Evidence level: Moderate
Consensus level: 90% (Strongly agree—50%; Agree with minor reservation—40%; Disagree with minor reservation—10%)

The diagnosis of GERD is generally made based on clinical symptoms, response to acid suppression, upper endoscopy, esophageal pH, and impedance monitoring. Diagnostic testing is usually not necessary in patients who present with typical symptoms, such as heartburn or acid regurgitation, or with mild–moderate GERD. Furthermore, pH and impedance testing equipment are not widely available in most areas of the SEA region. However, pH and impedance monitoring is recommended in GERD patients presenting with refractory symptoms.

Section 4: TREATMENT
Statement 11: In obese individuals, weight loss is recommended to improve control of GERD symptoms
Grade of recommendation: Strong
Evidence level: High
Consensus level: 100% (Strongly agree—80%; Agree with minor reservation—20%)

Evidence suggests a strong association between obesity and GERD.^52^, ^53^, ^54^A prospective cohort study (*n* = 332) found the prevalence of GERD symptoms to be high (37%) in overweight and obese subjects. As the participants lost weight, there was a significant decrease in the overall prevalence of GERD and the mean GERD symptom score.^55^ In another study (*n* = 15, 295), subjects with general or abdominal obesity had improvement in GERD symptoms with weight loss or decrease in circumference of the waist.^56^Also, it has been found that voluntary and controlled weight loss can reduce symptoms and the usage and dosage of PPIs in patients with GERD.^57^ In the Nord‐Trøndelag Health (HUNT) study, there was dose‐dependent association between weight reduction and improvement in gastroesophageal reflux symptoms as well as an increased likelihood of treatment success with anti‐reflux therapy.^58^

Statement 12: Avoidance of tobacco smoking and reduction of alcohol intake and modification of diet/lifestyle are important in the treatment of GERD
Grade of recommendation: Strong
Evidence level: Moderate
Consensus level: 100% (Strongly agree—80%; Agree with minor reservation—20%)

Dietary and lifestyle factors have been implicated as risk factors for GERD.^59^ In a Chinese study, 1581 participants completed symptom and lifestyle questionnaires. Some of the factors that were significantly associated with GERD included high body mass index (BMI), smoking, eating fast and beyond fullness, preference for spicy and high fat food, lying down soon after eating, and wearing girdles or corsets.^60^ Randomized controlled trials have shown that late evening meals increase time with supine acid exposure compared with early meals.^61^ A recent report revealed that short meal‐to‐bed time habit is a prominent risk factor of GERD symptoms during pregnancy.^62^

In the HUNT study, cessation of daily tobacco smoking improved gastroesophageal reflux symptoms from “severe” to “no” or “minor” complaints compared with persistent daily smoking in individuals using anti‐reflux medication at least weekly.^63^ While smoking has been associated with occurrence of GERD, there is no strong evidence for the association between alcohol consumption and GERD.^59^ In the absence of long‐term studies to assess the effect of alcohol on GERD, it seems to be more of a triggering or precipitating factor rather than a causal factor for GERD,^64^ and advising moderating ethanol use would be pragmatic advice a physician could offer.

Statement 13: Routine avoidance of specific foods and drinks is not recommended
Grade of recommendation: Strong
Evidence level: Moderate
Consensus level: 100% (Strongly agree—40%; Agree with minor reservation—60%)

There is little scientific evidence supporting the recommendation of avoiding particular foods. However, if certain foods, such as coffee, chocolate, peppermint, citrus, carbonated drinks, and spicy foods have been shown to trigger reflux for individual patients, then they should be avoided.^64^, ^65^ Furthermore, the correlation of ingesting certain foods with reflux symptoms often varies over time. With the diverse diet that the SEA population consumes, no conclusive statement can be made for avoidance of foods.

“Acidic” beverages are believed to worsen GERD, but in practice, correlation of symptoms is less clear.^66^ Similarly, a survey of 394 patients with heartburn noted a weak correlation between titratable acid content of popular beverages and symptoms.^67^ In case of carbonated beverages, physiological changes with ingestion are transient and have not been correlated with GERD symptoms as reported by a systemic review of 17 studies.^68^ A meta‐analysis showed no significant association among coffee intake, GERD symptoms, or mucosal disease.^69^

Statement 14: Elevation of the head of the bed is useful in improving nocturnal GERD symptoms
Grade of recommendation: Strong
Evidence level: Moderate
Consensus level: 90% (Strongly agree—60%; Agree with minor reservation—30%; Disagree with minor reservation—10%)

Patients with GERD are advised to sleep with head elevated as it has been found that the symptoms are more pronounced in supine position due to gravity.^59^ A study revealed that bed‐head elevation by 20 cm blocks reduced esophageal acid exposure and acid clearance time from baseline and led to some relief from heartburn and sleep disturbances.^70^ In a recent study, head elevation significantly reduced reflux symptoms and night‐time symptoms of patients treated on an outpatient basis with PPI compared with those sleeping without the elevation.^71^

Statement 15: Alginates should be considered for empirical treatment of patients with mild‐to‐moderate symptoms of acid reflux disease
Grade of recommendation: Strong
Evidence level: High
Consensus level: 100% (Strongly agree—80%; Agree with minor reservation—20%)

Alginates provide effective and immediate relief of reflux symptoms. In a systematic review, including 14 studies and 2095 subjects, alginate‐based therapies were found to be more effective in treating GERD symptoms as compared with placebo or antacids.^72^Alginate/antacids are also used for self‐medication to control the mild reflux disease.^73^

Dettmar *et al*. demonstrated an immediate therapeutic action with alginate (within 1 h of administration), which was considered faster in comparison to a PPI or an H_2_‐receptor antagonist (H_2_RA).^74^ Alginate was found superior to placebo and antacids for the treatment of mild GERD, and this monotherapy seems to be beneficial as an initial treatment.^46^ In the sodium alginate group, symptom resolution was observed to be higher, and the speed of action was faster in comparison to antacid group.^75^

Alginate‐based formulation was comparable to omeprazole in achieving a heartburn‐free period in patients with moderate episodic heartburn and is a promising alternative treatment for moderate GERD.^76^ In the most recent randomized clinical trial, an alginate‐containing product has been shown to be superior to antacid in post‐supper suppression of the acid pocket in obese individuals.^77^Additionally, the Turkish reflux study group consensus report recommended alginate monotherapy as an initial therapy for patients with mild GERD.^78^

Statement 16: PPIs are the mainstay of treatment for patients with persistent symptoms of GERD
Grade of recommendation: Strong
Evidence level: High
Consensus level: 100% (Strongly agree—100%)

The PPIs are currently the best drugs for the treatment of patients with true GERD. Relief of symptoms is usually good and prompt, particularly in patients with severe grades of esophagitis.^79^ However, many patients with reflux symptoms without a confirmed diagnosis of GERD respond poorly to treatment with PPIs.^79^

Statement 17: Alginates are a good adjunctive therapy for relief of GERD symptoms partially responsive to proton‐pump inhibitor therapy
Grade of recommendation: Strong
Evidence level: Moderate
Consensus level: 100% (Strongly agree—100%)

In Japanese patients with NERD, omeprazole when combined with sodium alginate resulted in complete resolution of heartburn for at least one full week compared with omeprazole alone.^80^ A randomized trial conducted in Taiwan showed that sodium alginate oral suspension was non‐inferior to omeprazole in the treatment of NERD patients.^81^ Another randomized, placebo‐controlled trial concluded that addition of an alginate‐containing product for individuals with partial response to PPI therapy improved their quality of life.^82^

It was recommended that at the primary care level, PPI or a combination of alginate–antacid and acid suppressive therapy can be administered together to patients presenting with symptoms of reflux, or ongoing symptoms incompletely controlled with acid suppressants.^83^ Alginate compounds are safe and easy to use. As an adjunctive therapy, these compounds have been shown to be useful in pregnancy. In a prospective study in pregnant women (*n* = 144) with symptoms of heartburn and/or reflux requiring treatment, Gaviscon Advance was effective in 91% of patients as assessed by the investigator and 90% as assessed by the patients.^84^ Alginate compounds have also been recommended for use in extra‐esophageal GERD as an adjunctive therapy, for example, in reflux‐associated laryngopharyngitis.^85^, ^86^

Statement 18: PPIs are generally safe drugs but should be used with caution when taken on a long‐term basis
Grade of recommendation: Strong
Evidence level: Moderate
Consensus level: 100% (Strongly agree—100%)

The PPIs are generally very safe drugs. However, several studies have brought up the issue of complications with long‐term use, which include risk of fractures, kidney injury, dementia, enterochromaffin‐like cell hyperplasia, gastric carcinoids, hypergastrinemia, osteoporosis, vitamin B‐12 deficiency, and others.^87^, ^88^, ^89^ Evidence supporting a causal association, however, has been weak, although caution is needed especially in at‐risk populations including the elderly. Furthermore, the dose or duration response of PPIs associated with adverse outcome(s) is unclear. In a recently published observational study, based on a large multicenter study on the use of PPIs together with aspirin and rivaroxaban, Moayyedi *et al*. only found a weak association with enteric infections.^90^ When indications for prescription are clear, PPIs should be used. Strategies for reducing the amount of PPI used in maintenance therapy for GERD patients include an on‐demand treatment or alternative day treatment.

## Discussion

In this first consensus on mild‐to‐moderate GERD in the SEA region, all statements achieved consensus agreement. Mild‐to‐moderate GERD is very commonly seen in clinical practice in the SEA region. The majority of cases are diagnosed when they present to doctors at the primary care and frequently do not undergo further investigations such as gastroscopy. Gastroscopy remains nonetheless a very important diagnostic test, especially in SEA countries where the background incidence of upper gastrointestinal cancers is high and cancers occur at a young age.^51^ More sophisticated and detailed investigations such as pH and impedance testing are not widely available in the SEA region and are also not required in the large majority of patients in this category. However, the role of several investigative tools has been gaining traction in the diagnosis of GERD as detailed in the “Lyon consensus” report and may have an important role to play in the future.^91^

**Figure 1:**
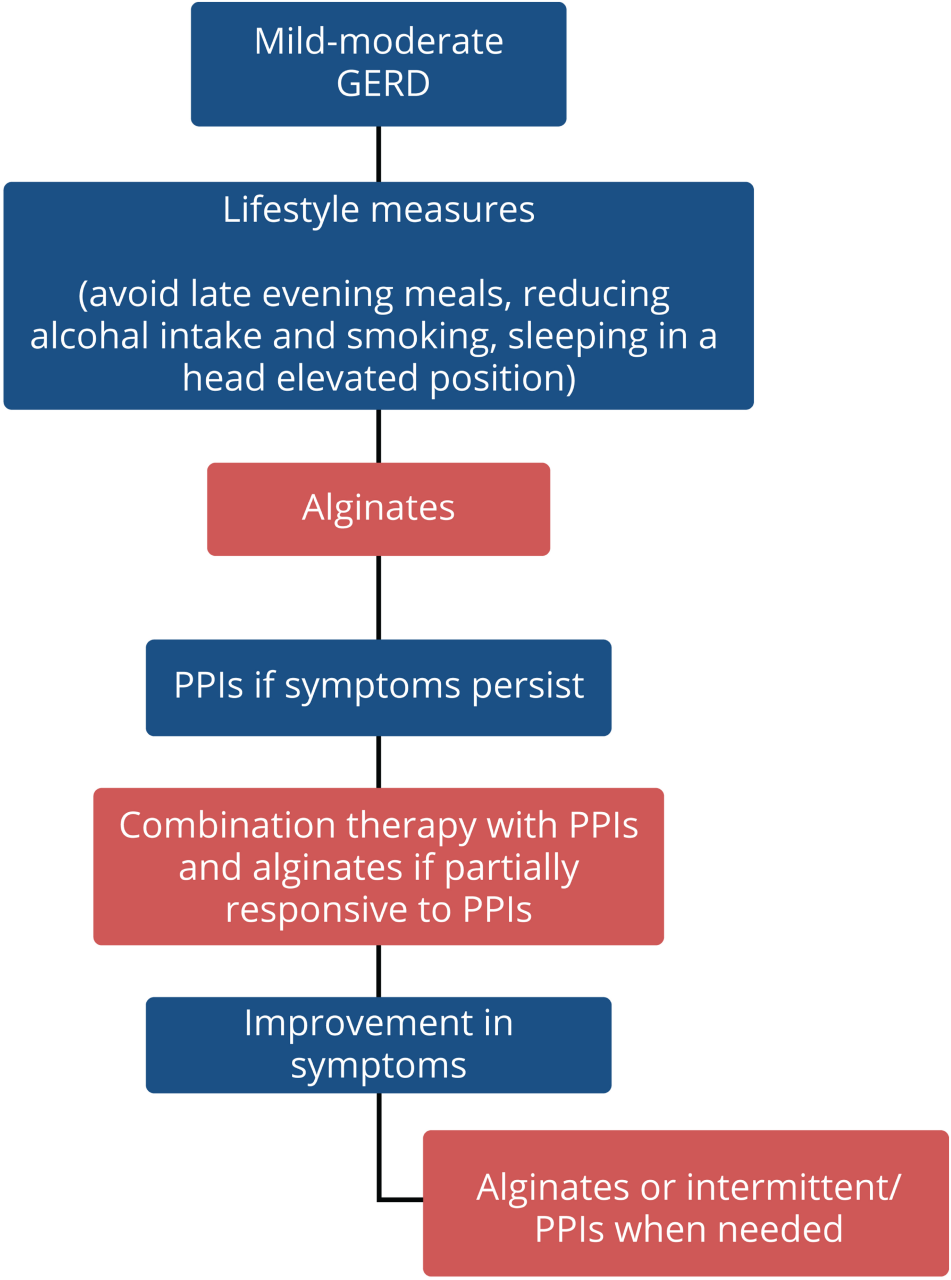
Algorithm for the management of mild‐to‐moderate gastroesophageal reflux disease (GERD) in the Southeast Asian region. PPIs, proton‐pump inhibitors.

Following the consensus process of discussion and voting, the expert panel has formulated a simple algorithm for the management of mild‐to‐moderate GERD (Fig. 1). In the management of patients with GERD, it is important to recommend lifestyle measures as the first step. Although, evidence may not be convincing for some lifestyle measures, it is reasonable to advice patients on weight loss and timing and volume of meals and head of the bed elevation.^58^, ^60^, ^61^, ^71^ These are simple measures that can be easily adopted by patients. Although PPIs have been commonly prescribed for the treatment of GERD, many patients, especially those who have been categorized as NERD, respond poorly to treatment with PPIs. Many of these patients may have functional heartburn or esophageal hypersensitivity.^30^ A new class of acid suppressing agents—the potassium channel acid blockers (PCABs)—have now been introduced for use in many countries in the Asia‐Pacific region. It has been shown to be more effective in the treatment of severe grades of erosive reflux esophagitis compared with PPIs but its role in the relief of symptoms in mild‐to‐moderate GERD has not been investigated.^92^

The concept of an acid pocket in the pathogenesis of acid reflux is now widely accepted.^36^ Alginates have a novel mechanism of action of forming barrier against reflux of this acid pocket.^46^ The expert panel has therefore recommended alginates as the first‐line empirical treatment of mild‐to moderate GERD.^72^, ^76^ Following the algorithm, PPIs are recommended for the treatment of patients with persistent symptoms of GERD.^79^ Additionally, alginates can be prescribed as an adjunctive therapy to PPIs in various circumstances such as during pregnancy^84^or even in extra‐esophageal GERD such as laryngopharyngitis or when patients are only partially responsive to PPIs.^85^, ^86^ Although alginates are a very safe compound for clinical use, there has to be some caution in use in patients with heart failure and chronic kidney diseases as they contain sodium or potassium. Excessive consumption outside prescribed dosages should be avoided.
